# Social Preference in Preschoolers: Effects of Morphological Self-Similarity and Familiarity

**DOI:** 10.1371/journal.pone.0145443

**Published:** 2016-01-04

**Authors:** Nadja Richter, Bernard Tiddeman, Daniel B. M. Haun

**Affiliations:** 1 Research Group for Comparative Cognitive Anthropology, Max Planck Institute for Evolutionary Anthropology, Leipzig, Germany; 2 Department of Developmental Psychology, University of Jena, Jena, Germany; 3 Department of Computer Science & Research Institute of Visual Computing, Aberystwyth University, Aberystwyth, United Kingdom; 4 Max Planck Institute for Psycholinguistics, Nijmegen, The Netherlands; 5 Department of Early Child Development and Culture, Leipzig University, Leipzig, Germany; University of Portsmouth, UNITED KINGDOM

## Abstract

Adults prefer to interact with others that are similar to themselves. Even slight facial self-resemblance can elicit trust towards strangers. Here we investigate if preschoolers at the age of 5 years already use facial self-resemblance when they make social judgments about others. We found that, in the absence of any additional knowledge about prospective peers, children preferred those who look subtly like themselves over complete strangers. Thus, subtle morphological similarities trigger social preferences well before adulthood.

## Introduction

Humans show homophilic tendencies when making social decisions. Among adults, shared similarities such as attitudes, opinions, lifestyle or even similar names or shared birthdates influence the selection of potential partners for mating and social interaction [1–5]. Further, facial resemblance is an important cue that promotes prosociality, trust and perceived attractiveness among adults [6–8] (but see [9]). For example, in economic games, participants allocated higher monetary contributions to unknown group members that subtly resembled themselves [10,11].

Here we focus on the early onset of responsiveness to self-similarity. Previous studies indicate that being similar predicts social preference already early in life. Children display biases based on shared social categories like gender, age, accent or ethnicity [12–15] and even rely on arbitrary similarity such as wearing randomly assigned markers or shirts of the same color [16]. Young children further favor others that match their own preferences in food or toy choices [17–19]. Hence, from early on, humans are guided by clearly marked and highly prominent cues when selecting among potential interaction partners. However, it is unknown whether the sensitivity to subtle, unmarked similarity found in adults is already present in childhood.

We here report a study that examined the effects of facial resemblance on children’s initial response towards individuals that they had never met before. The children thus lacked any information about the prospective interaction partner, except for what can be determined by looking at them. Based on previous work in adults, we expected that children would respond positively to subtle facial self-resemblance. Children were asked to select new classmates from pictures of unknown peers—a particularly salient scenario for 5-year-old German children anticipating their upcoming school enrollment. We adopted the method of “morphing”, a well-known tool in adult studies [8] to research with children. This technique allows for digitally altering facial resemblance in photographs of study participants. Faces can be mixed to create a composite novel face that resembles each of the input faces to a specified amount. We used this technique to create sets of stimulus faces for each of our participants. By comparing the subjects’ responses to morphed images that combine the subject’s own face to those that combine two strangers, we tested if children preferred self-similar peers.

To our knowledge this is the first study examining social assortment in childhood within the framework of morphological similarity of the face. We chose to study this in preschoolers specifically because it is not until around age 5 that children are sensitive to seemingly subtle and minimal similarities and show consistent tendencies to prefer those with whom they share even just a slight, seemingly arbitrary feature [16]. Thus, if children show preferences for minimal similarity at age 5, we might expect preferences based on subtle morphological similarity to be present around the same age.

One interpretation of the adult preference for self-similar faces is a subconscious detection of potential genetic relatedness [11]. Another explanation for such an effect might be that ones own face is a familiar stimulus and familiar stimuli have been shown to be preferred over novel stimuli in adults—the so-called mere exposure effect [20,21]. Although this effect is less stably found in children in comparison to adults [22], we aimed to include a control that would assess children’s preference for a face that subtly resembles a familiar face. We chose to operationalize familiarity in a minimalistic sense by exposing children to a photograph of a same-age, same-gender stranger several days before the testing session. The design therefore allowed us to examine the effects of similarity and minimal familiarity on social preference in children within a single test.

## Materials and Methods

### Ethics Statement

**Fig 1:**
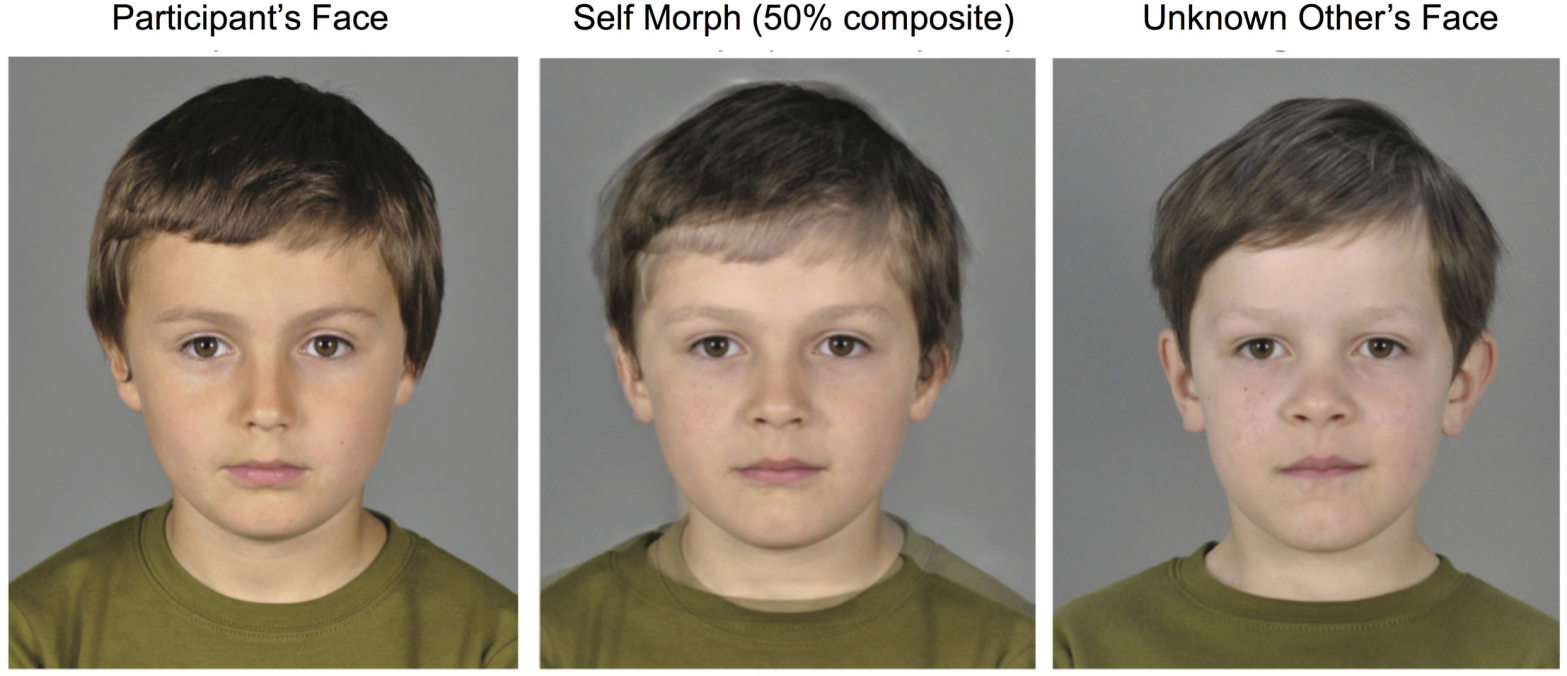
Facial resemblance manipulation. Shape and color information of the participant’s face (left) and a face of an individual unknown to the participant (right) were blended in a 50:50 ratio to create a composite face, the Self Morph (center). Only the internal face features have been morphed. The two individuals displayed here along with their legal guardians have given permission for their photographs to be used in this figure.

The study was conducted non-invasively and approved by the local ethics committee of the Max Planck Institute for Evolutionary Anthropology in Leipzig, Germany. Informed written consent was obtained from all parents of the participating children. Additionally, the individuals displayed in Fig 1 as well as their legal guardians have agreed and given written informed consent for their photographs to be published in a Public Library of Science (PLoS) Journal.

### Participants

Study participants were 5-year-old children (mean age = 5 years, 8 months, 15 days; range from *min* = 5;7;0 to *max* = 5;9;30) recruited from a database of parents from a medium-sized German city who had volunteered to participate in studies of child development. We decided in advance to collect data from 24 participants of each gender for both testing conditions (main & control, see below). Thus, a total of 96 children (48 female, 48 male) took part in the study, split evenly between the main condition and the control condition (see descriptions below). An additional six children were tested but excluded from the final sample because they either failed to remember a previously encountered face (see procedure details below; *n* = 2, main condition), or they detected, either spontaneously or prompted by a control question, that their own face was being used in the experiments (*n* = 2, main condition); i.e. self-detection instead of self-similarity detection. Two more children had to be excluded, one due to an experimenter error (control condition), and the other because the child did not give any responses (main condition). Seventy-six percent of the participants in the final sample had at least one sibling.

### Stimuli

We used image manipulation software [23] to create stimuli morphs. In each of 12 trials, the children were presented with a set of three pictures of age and gender matched morphs: an Unknown-, a Familiar- and a Self-Morph picture. The Self Morph was created by blending the facial features of the subject and those of a stranger, weighing each by 50% (Fig 1). Similarly, the Familiar Morph was created by blending a stranger with a “familiar” face, i.e. an individual whose picture the subject had encountered in the Familiarization Session (see procedure details below). Finally, the Stranger Morph was composed of two stranger faces. For each test trial, different input stranger faces were used in the respective Stranger-, Familiar-, and Self Morphs. All stranger faces were randomized across participants and matched in age and sex, guaranteeing distinctive stimuli.

All input faces were drawn from a database of ~250 standardized facial pictures of 5-year-old, German children of both sexes that we had created for the purpose of this research question. Faces in the database had previously been ranked on a 4-point Likert-type scale by 24 children (12 female) of the same age with respect to how “likeable” they appeared. We only used faces from the central 60% of this likeability distribution. The “Familiar Peers” (Nico) were two photographs per sex (interchanged across participants) with likeability equal to the median. Morphed stimuli were presented with masked contextual features in counterbalanced order and position across participants and trials on a 24-inch screen. All participating children had a neutral facial expression when photographed and scars or birthmarks were removed digitally (using Adobe Photoshop CS5). All study participants were white. In addition, all stimuli faces used for the morphing were white as well, thus all morphed faces were implicitly matched for skin color.

### Procedure

#### Main condition

Session 1: picture taking & familiarization. In the first session, children were photographed under the cover story that we needed many pictures of preschoolers to be used as team players in a virtual game. A general outline of the study was given to the accompanying parents and informed consent was obtained. Before their picture was taken, children were introduced to the Familiar Peer stimulus “Nico” (same name applied to both genders), a portrait photograph showing a child that matched the participant regarding age and sex. Along with the name, children were given the information that “Nico” had previously had her / his picture taken in the same way, and that this photograph would serve as a model for the facial expression they were asked to adopt. Children were then asked to hold on to that picture for 10 seconds while the experimenter pretended to write something down. In sum, the amount of exposure to the Familiar Peer stimulus’ face was about 15 to 20 seconds. This procedure produced almost perfect accuracy at recalling the familiarized face as only two out of 102 tested children failed to recognize Nico in the respective memory pretest during the testing session (98% success rate).

**Fig 2:**
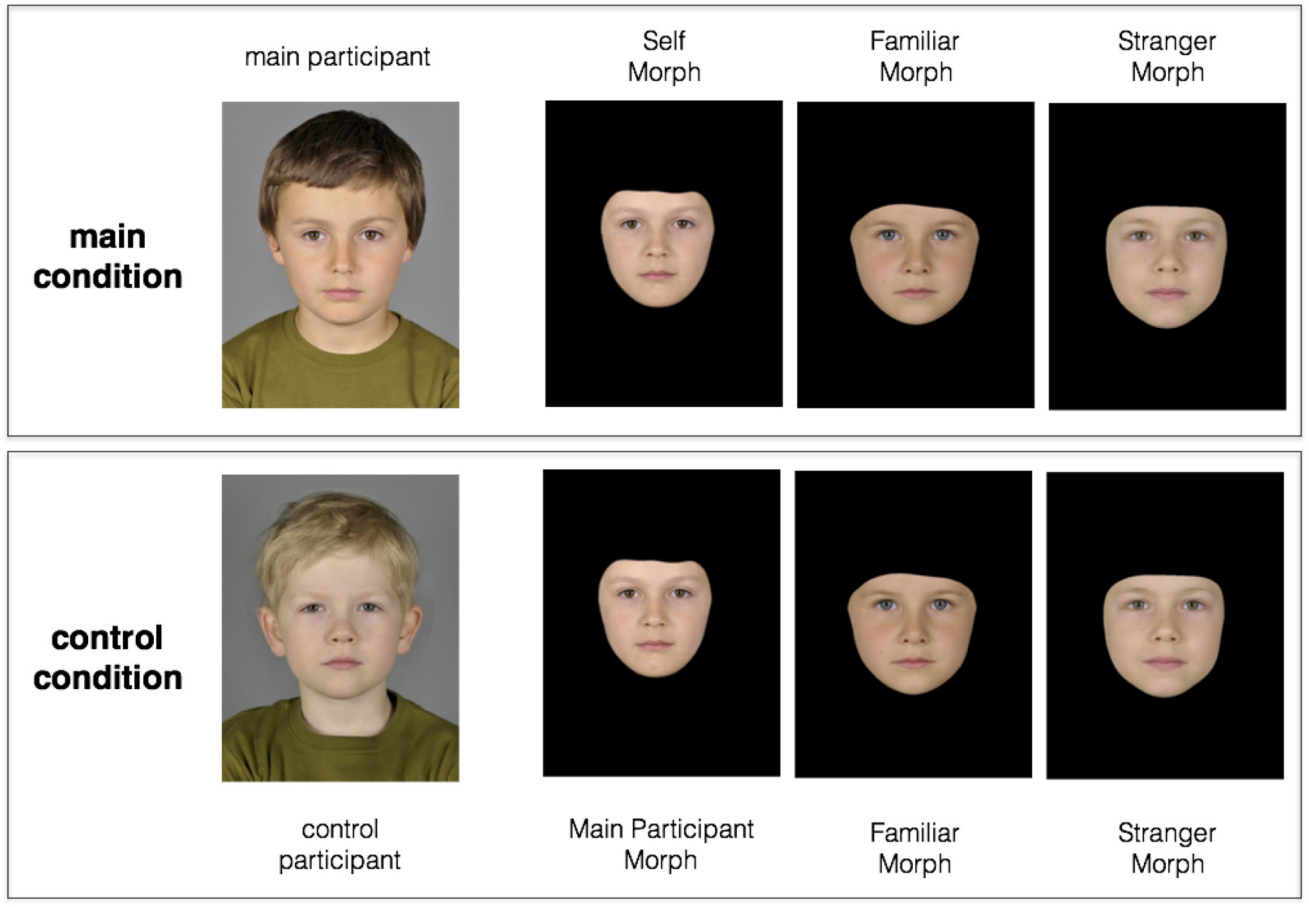
Overview of the experimental design. In each test trial, participants were presented with a set of three pictures of age and gender matched morphs. The masked pictures illustrate how stimuli were presented to the participating children in the test phase. The top row demonstrates the main testing condition where the participant’s face (top row, far left) was morphed with an unknown other’s face to create the Self Morph (top row, second left). Similarly, the Familiar Morph (top row, second right) was created by blending a stranger face with a previously encountered face while the Stranger Morph (top row, far right) was a composite of two stranger faces. Crucially, participants in the control condition (bottom row) were presented with the identical stimuli sets of a participant in the main condition. Therefore control participants never encountered self-similar stimuli but instead morphs that resembled the face of that other participant (bottom row, second left) who was not personally known to the control participant. Pairs of main and control participants had been randomly yoked together. The displayed control participant is not a real person but has been created for this illustration by digitally blending multiple faces.

Session 2: test. Children attended the study a few days after the Familiarization Session (mean delay = 3.6 days; range from *min* = 1 to *max* = 7). The study procedure took on average 15 minutes and included a memory pretest, followed by 12 test trials, control questions and 12 post test trials. Parents were not present during the test. For the memory pretest, children had to correctly identify Nico (the Familiar Peer) amongst a set of three portrait pictures of gender- and age-matched children (*“Do you recognize any of these children*?*”*). Next, they were similarly asked to indicate their own portrait picture amongst two other age- and gender-matched strangers. In order to pass the memory test, children had to correctly point out the pictures of Nico and themselves on the first try. All children accurately identified their own facial portrait. The two children who failed to recall Nico’s face were not included in the final data set. Children were then told that they were going to look at several rounds of pictures of peers on the screen and that they had to decide and point at whom they would most like to have as new classmates in school next year. Twelve successive test trials were presented. On each trial, three stimuli (one Familiar-, one Self- and one Stranger Morph) appeared in centered juxtaposition on the screen and children were asked to point at their preferred choice (see Fig 2 for an illustration how stimuli were presented).

After completion of test trials, participants saw the same stimuli again and were asked to indicate if they knew or had ever seen the ‘children’ being shown before (control questions). This was done to ensure that the children did not recognize (correctly or incorrectly) any of the morphed images as a person they knew. For the last part of the study, 12 post test trials with the same stimuli were presented in identical forced choice manner and participants had to indicate which of the stimuli resembled themselves most (“*Which one looks most similar to yourself*?”). Children and parents were debriefed by the experimenter and informed of the study’s motivations. All parts of the experimental procedure were recorded on video and children’s responses were coded both in situ and from video.

#### Control condition

Because the face of the subject recurs in each trial (morphed with a different child in each case), the subject may develop a preference simply based on repeated exposure to some facial features that recur across Self Morphs. We therefore conducted a control experiment with the same sample size, in which participants never encountered self-resembling stimuli (between-subject condition; also see Fig 2 for an overview of the experimental design). Instead, they were presented with the stimulus set of a different participant. The ‘Self Morphs’ in these sets therefore resembled the face of that different participant (who was not personally known to the control participant), morphed with 12 different stranger peers. The procedure of the control condition was identical to that of the main condition with two exceptions. First, control participants were not photographed and therefore did not have to identify themselves in a memory pretest (instead, they saw a picture of the subject whose stimulus set was used). Second, in the final section of the test, children were asked which of the stimuli mostly resembled Nico, the Familiar Peer, instead of themselves (“Which one looks most similar to Nico?”). Identical to the experimental condition, participants had been familiarized with Nico’s picture in a session prior to the test.

### Data analysis

On each of 12 test trials, we presented three stimuli (Self / Main Participant Morph vs. Familiar Morph vs. Stranger Morph) in a forced choice manner and coded the participant’s choices. Hence, we collected repeated responses per subject (one per trial). Further, two out of 50 tested children in the main condition detected our experimental manipulation and recognized that their own face was used in the Self Morph stimuli. This led to an exclusion of these cases. Including these cases in our analysis did not alter the results (S1 Fig).

Our initial analysis was aimed at testing whether children varied in their choices between the three different stimuli types. Our further strategy of data analysis was aimed at testing single predictions that we based on the following rationales:

1. *Children prefer self-similar others over strangers*. Under this prediction we expected that (a) participants in the main condition choose the Self Morph stimuli more often than the participants in the control condition (because, crucially, for them it resembles a stranger, i.e. the main participant). Therefore, our target analysis was focused on choices of the Self Morph stimuli in the Main Group versus choices of that identical stimulus (Main Participant Morph) in the Control Group. Furthermore, we expected that (b) Self Morphs are chosen more frequently than Stranger Morphs in the main condition.
2. *Children prefer familiar others over strangers*. Under this prediction we expected that Familiar Morphs are chosen more frequently than Stranger Morphs. Since participants received identical information and exposure to Nico, the Familiar Peer in both testing conditions we should see (a) preferences of Familiar- over Stranger Morphs in the Main condition and the Control condition. We also expected (b) preferences of Familiar- over Main Participant Morphs and Stranger Morphs in the control condition.
3. *Children prefer self-similar over familiar others*. Under this prediction we expected that (a) participants select Self Morphs more frequently than Familiar Morphs in the main condition. We further expected that (b) if self-similarity were preferred we should see less Familiar Morph choices in the presence of the “competing” Self Morph (main condition), but more Familiar Morph choices in the absence of the “competing” Self Morph (control condition).

Within our target analysis we applied non-parametric tests including unequal variance t-tests based on rank transformations of the data, which we chose according to their rationales and assumptions [24–26]. Tests of small samples were exact [25,27]. All reported p-values are two-tailed. We performed all analysis using R 3.1.0 [28], and for some tests the R packages *exactRankTests* [29] and *gtools* [30].

## Results

### Main analyses

We found no differences in the responses to the Self Morph between sexes, neither in the main condition (Welch Two Sample t-test: *t* = 0.311, *df* = 44.90, *p* = 0.757), nor in the control condition (Welch Two Sample t-test: *t* = 0.690, *df* = 44.83, *p* = 0.494), and hence pooled responses from boys and girls (*n* = 48 per condition).

**Fig 3:**
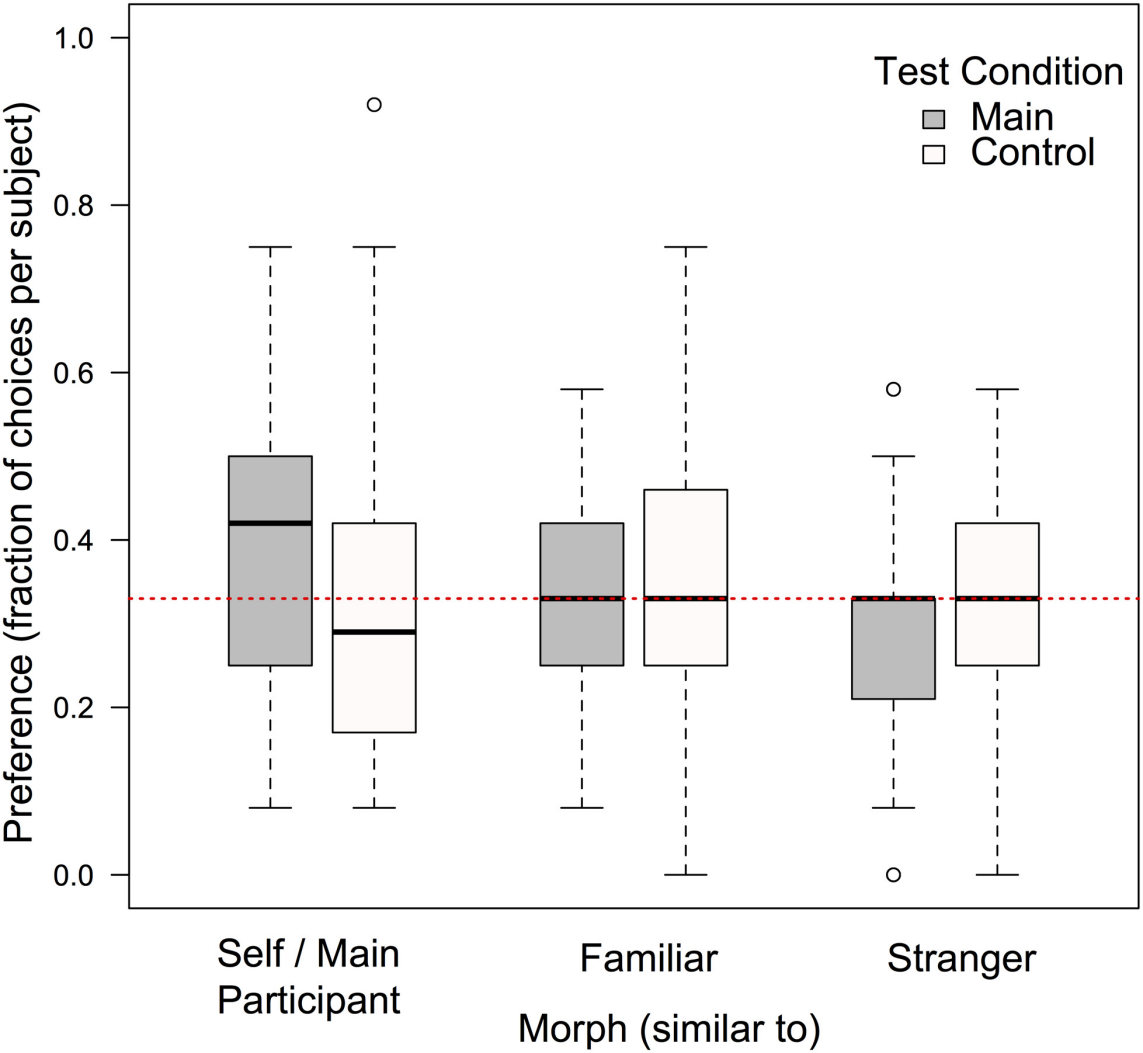
Response distribution demonstrating 5-year-Olds’ preferred choices. The box-and-whisker plots show children’s responses across all three stimuli types in the two experimental conditions. Only in the main condition (gray boxes, n = 48) did the Self Morph resemble the participant’s face, while for participants in the control condition (white boxes, n = 48), these stimuli resembled a control face from another, unfamiliar participant. The boxes indicate the first and fourth quartiles. The solid lines inside the boxes represent the medians. The dashed lines capture the location of extreme values, with the exception of outliers (shown as circles) that exceeded the inter-quartile distance by more than 1.5. The horizontal, red dashed line indicates the chance level (33%).

There was no overall difference among responses across the three stimuli types in both testing conditions (Friedman χ^2^_MainGroup_ = 4.1, *df* = 2, *p* = 0.13; Friedman χ^2^_ControlGroup_ = 2.77, *df* = 2, *p* = 0.25, see Fig 3).

1. *Do children prefer self-similar others over strangers?* Children in the main condition chose their own Self Morphs more frequently than children in the control condition chose the identical stimulus, i.e. the Main Participant Morph (Welch Two Sample t-test: *t* = -2.076, *df* = 93.24, *p* = 0.041; Fig 3). Because our prediction was a preference for the Self Morph over the Stranger Morph in the main condition, we directly compared these two stimuli despite the absence of an overall effect. Children chose the Self Morphs significantly more frequently than the Stranger Morphs (exact Wilcoxon test: *T*^+^ = 728, *N* = 46 (2 ties), *p* = 0.040). Children opted for the Self Morphs in a median of 42% of trials, which does not deviate from what is expected by chance (Exact Wilcoxon signed rank test: *T*^+^ = 620, *N* = 48, *p* = 0.074). In the control group, the corresponding identical stimuli (Main Participant Morphs) were similarly chosen as expected by chance, in a median of 29% of trials (Exact Wilcoxon signed rank test: *T*^+^ = 294, *N* = 48, *p* = 0.389).

2. *Do children prefer familiar others over strangers?* Pairwise comparisons indicated that while children in the main condition chose the Familiar Morphs more frequently than the Stranger Morphs (exact Wilcoxon test: *T*^+^ = 668, *N* = 44 (4 ties), *p* = 0.043), children in the control condition did not differ in their choices between these two (exact Wilcoxon test: *T*^+^ = 462, *N* = 42 (6 ties), *p* = 0.9). There were further no differences in the frequency of choices between Familiar Morphs and Main Participant Morphs in the control condition (exact Wilcoxon test: *T*^+^ = 588, *N* = 44 (4 ties), *p* = 0.28). Familiar Morph choices in both conditions did not differ from chance expectation (exact Wilcoxon signed rank tests, all *p*s > 0.12).

3. *Do children prefer self-similar over familiar others?* There were no differences in the frequencies of choices between Self Morphs and Familiar Morphs in the main condition (exact Wilcoxon test: *T*^+^ = 518.5, *N* = 43 (5 ties), *p* = 0.597). Children in both conditions opted for the Familiar Morph in similar ways, i.e. they chose that stimulus in a median 33% of trials (range_MainGroup_ = 0.08–0.58 & range_ControlGroup_ = 0–0.75 per subject; Welch Two Sample t-test: *t* = -0.045, *df* = 93.12, *p* = 0.97; Fig 3).

### Additional analyses (post test trials)

As responses on post test trials did not differ between sexes (Welch Two Sample t-tests: *t*_Main Condition_ = 1.37, *df* = 45.98, *p* = 0.177 & *t*_Control Condition_ = 0.19, *df* = 45.50, *p* = 0.854), data were again pooled.

We found that children were able to recognize self-similarity in our experimental manipulation better than expected by chance (Exact Wilcoxon signed rank test: *T+* = 928, *N* = 48, *p* < 0.001). More specifically, children correctly identified self-similarity in the Self Morphs in a median 58% of trials (range = 0.08 to 1.0 per subject; main condition), while 33% are expected randomly.

Crucially, for control participants no actual self-similarity was present in any of the presented stimuli, so we here asked participants to indicate similarity to the Familiar Peer “Nico”. We observed that children in the control condition correctly identified resemblance to a previously encountered face in a median 50% of trials (range = 0.17 to 0.92 per subject) which is significantly higher than chance expectation (Exact Wilcoxon signed rank test: *T+* = 807, *N* = 48, *p* < 0.004; control condition).

## Discussion

Previous studies have found prosocial effects triggered by facial self-resemblance in adults, yet it is not known whether sensitivity towards a subtle cue of self-similarity guides social assortment already at an early age. In the current study, 5-year-old preschoolers had to choose their preferred classmate without knowing that aspects of their own face had been mixed into the faces of some of the pictured peers. We found that they favored peers who resembled themselves over those composed of two unknown strangers (prediction 1b). This preference was not simply due to low-level visual features of the stimuli, since children who did not share any facial features with those same images did not prefer them (prediction 1a).

In regard to the question whether children in our study also preferred faces that subtly resembled a face that had been exposed twice before over morphs composed of novel faces, our conclusions are less clear. While one of our predictions was partially met (2a) the other could not be confirmed (2b). While the children in the main condition preferred faces that subtly resembled a face that had been exposed twice before over morphs composed of novel faces, children in the control condition did not prefer the familiar faces over any of the two alternatives that were both composed of strangers. Hence we cannot come to a firm conclusion based on the present data. It is possible that children’s preference for self-similar others stems from familiarity with their own face. Self Morphs are by definition self-similar as well as highly familiar. Thus the more stable preference for Self Morphs in contrast to Familiar Morphs in our study might be due to higher familiarity with one’s own face, in addition to the minimal familiarity attached to „Nico“. Disentangling the impact of self-similarity and familiarity preferences would need to contrast self-similar faces with equally familiar, yet not self-similar, control faces.

Our experiment examines ontologically early social preference in the absence of any reputational information or learning. We showed that children, as has been claimed for adults, prefer similar others based on a subconscious assessment of similarity in facial features. This effect could be routed in kinship detection mechanisms [11] as well as a preference for similarity and familiarity because this indicates a common ontogenetic context, which in turn simplifies coordination in joint activities and increases predictability with similar hence more predictable others [31,32]. Our data further show that children correctly identified self-similar faces more frequently (in a post test, they succeeded 58% of the time) than they had preferred the identical faces during initial exposure (42% of the time). A more detailed analysis of whether similarity recognition influences children’s preferences is problematic because choices in the initial test trials may confound subsequent choices due to order effects. However, we can conclude that the correspondence between detection of self-similarity and preference is not perfect—children reliably detect facial resemblance but do not always use this information in their social preferences.

Future studies should aim to describe the specific cognitive mechanisms underlying the effects of self-similarity and familiarity preferences. Previous work has suggested automatic learning generalization processes might underlie the evaluation of novel faces in adults and children [33].

In summary, we provide the first evidence that the tendency to select social partners based on facial self-resemblance, a subtle cue of kinship [7,10,34] begins well before adulthood. Thus, preference for facial similarity is a strong guide to social assortment, especially when any other useful information about the potential interaction partner is missing.

## Supporting Information

S1 Dataset Data collected in both testing conditions (between-subject design). Note that this dataset comprises responses from the two participants that were excluded from final data analysis due to detecting the experimental manipulation (see S1 Fig).
(XLS)

S1 Fig Response distribution including data of the two participants who detected the experimental manipulation during testing, i.e. they detected their own faces in stimuli morphs. The box-and-whisker plots show children’s responses across all three stimuli types in the two experimental conditions. Only in the main condition (gray boxes, n = 50) did the Self Morph resemble the participant’s face, while for participants in the control condition (white boxes, n = 48), these stimuli resembled a control face from another, unfamiliar participant. The boxes indicate the first and fourth quartiles. The solid lines inside the boxes represent the medians. The dashed lines capture the location of extreme values, with the exception of outliers (shown as circles) that exceeded the inter-quartile distance by more than 1.5. The horizontal, red dashed line indicates the chance level (33%).
(TIF)

## References

[1] Berscheid E, DionElaine K, Walster GW. Physical attractiveness and dating choice: A test of the matching hypothesis. J Exp Soc Psychol. 1971;7: 173–189.

[2] Byrne DE. The attraction paradigm. Academic Pr; 1971.

[3] Gruenfeld DH, Tiedens LZ. Organizational preferences and their consequences. Handb Soc Psychol. 2010;3:III:33.

[4] Jones JT, Pelham BW, Carvallo M, Mirenberg MC. How Do I Love Thee? Let Me Count the Js: Implicit Egotism and Interpersonal Attraction. J Pers Soc Psychol. 2004;87: 665–683. 10.1037/0022-3514.87.5.665 15535778

[5] McPherson M, Smith-Lovin L, Cook JM. Birds of a feather: Homophily in social networks. Annu Rev Sociol. 2001; 415–444.

[6] Bailenson JN, Iyengar S, Yee N, Collins NA. Facial Similarity between Voters and Candidates Causes Influence. Public Opin Q. 2009;72: 935–961. 10.1093/poq/nfn064

[7] Bressan P, Zucchi G. Human kin recognition is self- rather than family-referential. Biol Lett. 2009;5: 336–338. 10.1098/rsbl.2008.0789 19324640 PMC2679920

[8] DeBruine LM, Jones BC, Little AC, Perrett DI. Social Perception of Facial Resemblance in Humans. Arch Sex Behav. 2007;37: 64–77. 10.1007/s10508-007-9266-0 18157627

[9] Giang T, Bell R, Buchner A. Does Facial Resemblance Enhance Cooperation? Sorci G, editor. PLoS ONE. 2012;7 10.1371/journal.pone.0047809 PMC3477107 23094095

[10] DeBruine LM. Facial resemblance enhances trust. Proc R Soc B Biol Sci. 2002;269: 1307–1312. 10.1098/rspb.2002.2034 PMC1691034 12079651

[11] Krupp D, DeBruine LM, Barclay P. A cue of kinship promotes cooperation for the public good. Evol Hum Behav. 2008;29: 49–55. 10.1016/j.evolhumbehav.2007.08.002

[12] Cohen E, Haun D. The development of tag-based cooperation via a socially acquired trait. Evol Hum Behav. 2013;34: 230–235. 10.1016/j.evolhumbehav.2013.02.001

[13] Jacklin CN, Maccoby EE. Social behavior at thirty-three months in same-sex and mixed-sex dyads. Child Dev. 1978; 557–569.

[14] Kinzler KD, Dupoux E, Spelke ES. The native language of social cognition. Proc Natl Acad Sci. 2007;104: 12577 17640881 10.1073/pnas.0705345104 PMC1941511

[15] Kinzler KD, Shutts K, Correll J. Priorities in social categories. Eur J Soc Psychol. 2010;40: 581–592. 10.1002/ejsp.739

[16] Dunham Y, Baron AS, Carey S. Consequences of “Minimal” Group Affiliations in Children. Child Dev. 2011;82: 793–811. 10.1111/j.1467-8624.2011.01577.x 21413937 PMC3513287

[17] Fawcett CA, Markson L. Similarity predicts liking in 3-year-old children. J Exp Child Psychol. 2010;105: 345–358. 10.1016/j.jecp.2009.12.002 20092828

[18] Hamlin JK, Mahajan N, Liberman Z, Wynn K. Not Like Me = Bad: Infants Prefer Those Who Harm Dissimilar Others. Psychol Sci. 2013;24: 589–594. 10.1177/0956797612457785 23459869 PMC4374623

[19] Mahajan N, Wynn K. Origins of “Us” versus “Them”: Prelinguistic infants prefer similar others. Cognition. 2012;124: 227–233. 10.1016/j.cognition.2012.05.003 22668879

[20] Zajonc RB. Attitudinal effects of mere exposure. J Pers Soc Psychol. 1968;9: 1–275667435

[21] Zajonc RB. Mere Exposure: A Gateway to the Subliminal. Curr Dir Psychol Sci. 2001;10: 224–228. 10.1111/1467-8721.00154

[22] Bornstein RF. Exposure and Affect: Overview and Meta-Analysis of Research, 1968–1987. Psychol Bull. 1989;106: 265–289.

[23] Tiddeman B, Burt M, Perrett D. Prototyping and transforming facial textures for perception research. Comput Graph Appl IEEE. 2001;21: 42–50.

[24] Ruxton GD. The unequal variance t-test is an underused alternative to Student’s t-test and the Mann-Whitney U test. Behav Ecol. 2006;17: 688–690. 10.1093/beheco/ark016

[25] Siegel S, Castellan N. Nonparametric statistics for the behavioral sciences. McGraw-Hill; 1988.

[26] Zimmerman DW. A note on consistency of non-parametric rank tests and related rank transformations. Br J Math Stat Psychol. 2012;65: 122–144. 10.1111/j.2044-8317.2011.02017.x 21518333

[27] Mundry R, Fischer J. Use of statistical programs for nonparametric tests of small samples often leads to incorrect P values: examples from Animal Behaviour. Anim Behav. 1998;56: 256–259. 9710485 10.1006/anbe.1998.0756

[28] R Core Team. R: A Language and Environment for Statistical Computing. Vienna, Austria: R Foundation for Statistical Computing; 2015 Available: http://www.R-project.org/.

[29] Hothorn T, Hornik K. exactRankTests: Exact Distributions for Rank and Permutation Tests. 2015. R package version 0.8–28. Available: http://CRAN.R-project.org/package=exactRankTests.

[30] Warnes GR, Bolker B, Lumley T. gtools: Various R programming tools. 2015. R package version 3.5.0. Available: http://CRAN.R-project.org/package=gtools.

[31] Haun DB, Over H. Like me: a homophily-based account of human culture Cultural evolution, a Strungmann Forum Report. Cambridge, MA: MIT Press; 2013.

[32] Heyes C. What Can Imitation Do for Cooperation? In: Sterelny K, Joyce R, Calcott B, Fraser B, editors. Cooperation and its evolution. Cambridge, MA: MIT Press; 2013 pp. 313–332.

[33] Verosky SC, Todorov A. When physical similarity matters: Mechanisms underlying affective learning generalization to the evaluation of novel faces. J Exp Soc Psychol. 2013; 10.1016/j.jesp.2013.02.004

[34] Maloney LT, Dal Martello MF. Kin recognition and the perceived facial similarity of children. J Vis. 2006;6. 10.1167/6.10.4 17132076

