# Supplementary figures and images for: Social Preference in Preschoolers: Effects of Morphological Self-Similarity and Familiarity
Source: PLoS One. 2016 Jan 4;11(1):e0145443. doi: 10.1371/journal.pone.0145443 (PMC4699649; doi:10.1371/journal.pone.0145443)

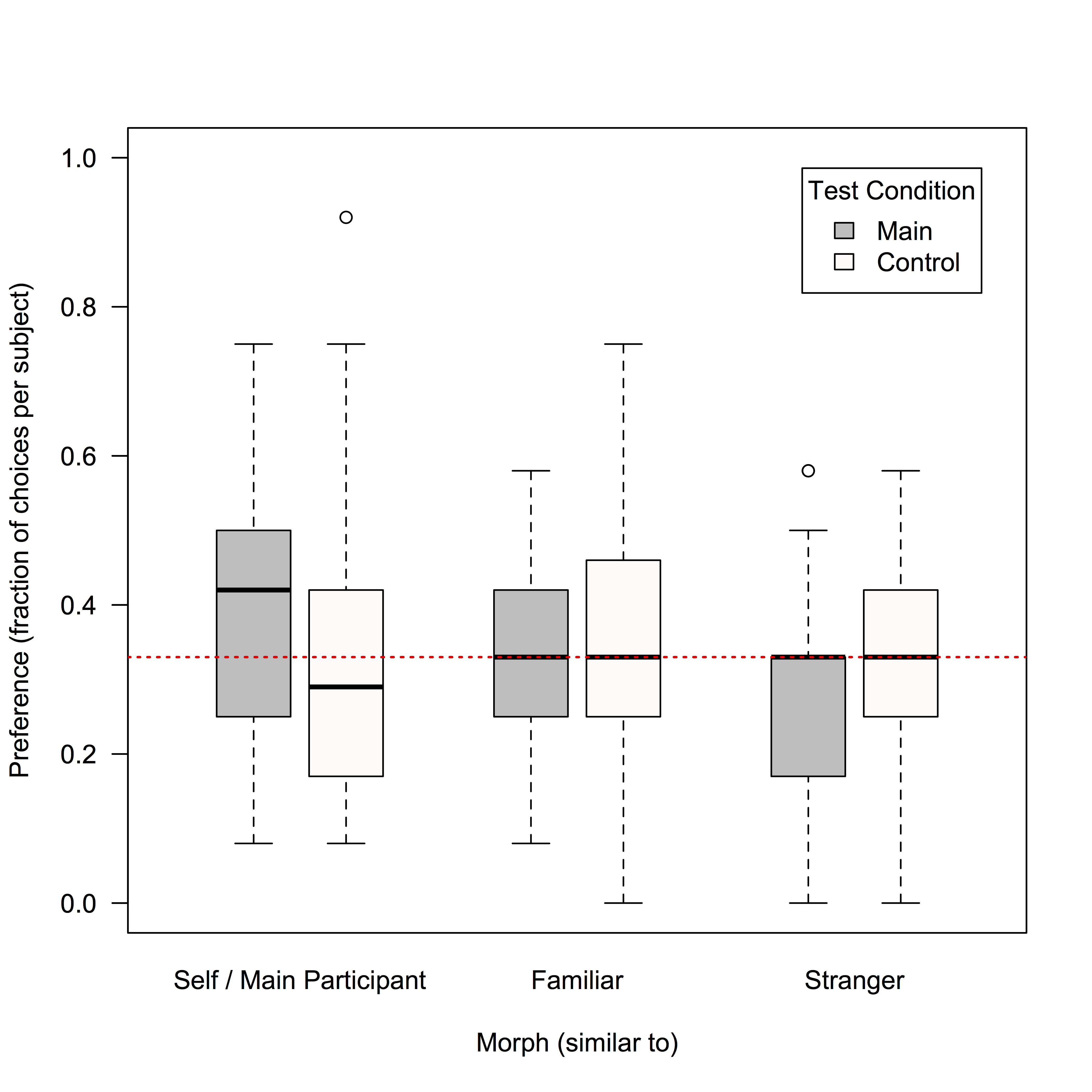

Supplement: S1 Fig — The box-and-whisker plots show children’s responses across all three stimuli types in the two experimental conditions. Only in the main condition (gray boxes, n = 50) did the Self Morph resemble the participant’s face, while for participants in the control condition (white boxes, n = 48), these stimuli resembled a control face from another, unfamiliar participant. The boxes indicate the first and fourth quartiles. The solid lines inside the boxes represent the medians. The dashed lines capture the location of extreme values, with the exception of outliers (shown as circles) that exceeded the inter-quartile distance by more than 1.5. The horizontal, red dashed line indicates the chance level (33%). (TIF) [file pone.0145443.s002.tif]
